# Annealing-Modulated Surface Reconstruction for Self-Assembly of High-Density Uniform InAs/GaAs Quantum Dots on Large Wafers Substrate

**DOI:** 10.3390/nano13131959

**Published:** 2023-06-28

**Authors:** Xiangjun Shang, Xiangbin Su, Hanqing Liu, Huiming Hao, Shulun Li, Deyan Dai, Mifeng Li, Ying Yu, Yu Zhang, Guowei Wang, Yingqiang Xu, Haiqiao Ni, Zhichuan Niu

**Affiliations:** 1 State Key Laboratory of Superlattices and Microstructures, Institute of Semiconductors, Chinese Academy of Sciences, Beijing 100083, China; xjshang@semi.ac.cn (X.S.); hqliu@semi.ac.cn (H.L.); lishulun@semi.ac.cn (S.L.); dydai@semi.ac.cn (D.D.); zhangyu@semi.ac.cn (Y.Z.);; 2 Center of Materials Science and Optoelectronics Engineering, University of Chinese Academy of Sciences, Beijing 100049, China; 3 Beijing Academy of Quantum Information Sciences, Beijing 100193, China; 4 State Key Laboratory of Optoelectronic Materials and Technologies, Sun Yat-sen University, Guangzhou 510275, China; yuying26@mail.sysu.edu.cn

**Keywords:** self-assembled quantum dots, deposition, migration, nucleation, nucleation site, quantum dot density, photoluminescence spectrum, full width at half maximum, in situ annealing, surface arsenic, reconstruction phase, quantum dot size distribution, T-dependent state population, growth rate, growth temperature, deposition amount, arsenic influence

## Abstract

In this work, we developed pre-grown annealing to form β2 reconstruction sites among β or α (2 × 4) reconstruction phase to promote nucleation for high-density, size/wafer-uniform, photoluminescence (PL)-optimal InAs quantum dot (QD) growth on a large GaAs wafer. Using this, the QD density reached 580 (860) μm^−2^ at a room-temperature (T) spectral FWHM of 34 (41) meV at the wafer center (and surrounding) (high-rate low-T growth). The smallest FWHM reached 23.6 (24.9) meV at a density of 190 (260) μm^−2^ (low-rate high-T). The mediate rate formed uniform QDs in the traditional β phase, at a density of 320 (400) μm^−2^ and a spectral FWHM of 28 (34) meV, while size-diverse QDs formed in β2 at a spectral FWHM of 92 (68) meV and a density of 370 (440) μm^−2^. From atomic-force-microscope QD height distribution and T-dependent PL spectroscopy, it is found that compared to the dense QDs grown in β phase (mediate rate, 320 μm^−2^) with the most large dots (240 μm^−2^), the dense QDs grown in β2 phase (580 μm^−2^) show many small dots with inter-dot coupling in favor of unsaturated filling and high injection to large dots for PL. The controllable annealing (T, duration) forms β2 or β2-mixed α or β phase in favor of a wafer-uniform dot island and the faster T change enables optimal T for QD growth.

## 1. Introduction

Self-assembled InAs quantum dots (QDs) have been used in optoelectronic devices, such as laser diodes [1], super-luminescence diodes [2], quantum emitters [3] and photodetectors [4], with flexible wavelength tuning by metamorphic growth [5,6,7], compatibility with silicon for integration [1,8,9], high working temperatures (T) [10] and speed [11]. Molecular beam epitaxy with simple nucleation mechanism, ultra-high vacuum and precise shutter could fabricate defect-free QDs with exciton formed [12]. For mass production of these devices, a large substrate with homogeneous island is desired. However, different Ts in the wafer center and surrounding (‘Surr’) show diverse migration; besides, the QD self-assembly has a continuous size distribution and critical coverage for large dots [13]. The QD density increases monotonously as the In deposition rate raises (932 μm^−2^ at the highest) [14] or using Al or Sb atoms [15,16] as preferential nucleation sites (>1000 μm^−2^). The strain sites in bilayer QDs also form uniform QDs (110 μm^−2^) in photoluminescence (PL) spectral full width at half maximum (FWHM) of ~17.5 meV [10]. Around the QD growth T, the GaAs (001) surface has many arsenic-rich (2 × 4) reconstruction phases: α, β, β2 and γ [17], with different influences on In adatom migration and nucleation. After GaAs growth, the γ phase with arsenic dimers in favor of cluster formation must be reduced for the QD island, usually, by ‘stay-by’ at the growth T in lower arsenic pressure for minutes, to form β with ordered surface arsenic in favor of In migration (T-sensitive). In this work, we use in situ annealing to form β2 nucleation sites for a uniform QD island at a spectral FWHM of ~23.6 meV (110~180 μm^−2^); an α phase with sufficient migration forms QDs in a wafer-uniform high PL at FWHM of 24~25 meV; a β phase at a high rate forms a QD density of 580~820 μm^−2^ with wafer-uniform PL spectra (i.e., large dots) (FWHM: 34~41 meV, as in [14]); a mediate rate forms uniform-size QDs in β (density: 320~400 μm^−2^, FWHM: 28~34 meV) while diverse QDs are formed in β/β2 (density: 370~440 μm^−2^, FWHM: 92~68 meV) with island in advance. Based on the PL behaviors, a physical picture of nucleation is obtained: The annealing forms β2-mixed α or β phase on a 3-inch wafer for a uniform island. The mediate annealing forms more β2 sites to limit migration while high (low) annealing forms an α (β) phase to promote it. The α phase with a Ga-Ga bond is in favor of In migration; the arsenic bond to Ga breaks the Ga-Ga bond and desorbs the Ga-As molecule to transfer α to β2 (i.e., the nucleation site) in the lower-T region while forming a Ga-As-Ga bond (i.e., direct α->β transfer) in the higher-T region with In migration unaffected. The QD growth parameters were optimized and the QD height distribution was analyzed. PL spectra at a low T or strong excitation reflect photocarrier populations in discrete states. The ground state in QDs shows a thermal activation energy of 0.24 eV (as the T-dependent PL spectra reflect), in favor of a high-T work. High-density QDs with a little-filled excited states are desired for high injection and optimal emission. The annealing offers a flexible tune of reconstruction phase, dependent on the annealing T, duration and arsenic pressure.

## 2. Experimental Section

**Figure 1:**
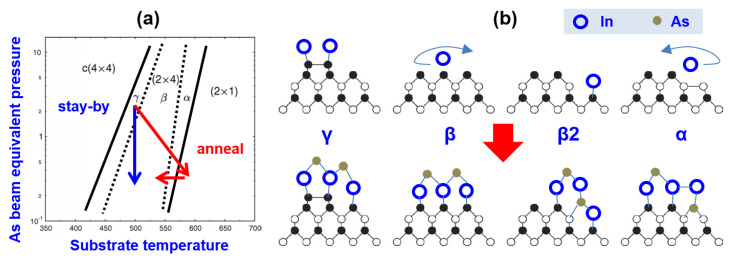
(**a**) *Arsenic pressure-T* GaAs(001) reconstruction phase diagram, two transfer indicated. (**b**) Structure model of arsenic-rich (2 × 4) reconstruction phases [17] and schematic of In migration and nucleation on them. γ at the lowest T with arsenic dimer tends to bond In adatom to form clusters; β with ordered arsenic promotes In migration; β2 with atomic stage is in favor of In adatom bond to form tall QD; α at higher T with Ga-Ga bond and less surface arsenic promotes In migration and enables easy transfer to β2 with arsenic (remove Ga atom in Ga-Ga bond) or to β in higher T (form Ga-As-Ga bond), for wafer-uniform island. The deposition of sub-ML Ga will form Ga-rich (4 × 6) reconstruction [19]. At T > 580 °C, there is Ga-rich (2 × 1) reconstruction [20] where In desorption is great, hard to island.

The QDs were grown on a 3-inch N^+^ GaAs(001) substrate in a solid-source molecular beam epitaxy. The epi-ready substrate was directly fixed on the standard holder with sapphire wafer at its backing for uniform heating. The QD growth was fulfilled as follows: After deoxidizing at 690 °C, the substrate was cooled to 630 °C to grow a 300 nm GaAs buffer at a rate of 0.6 μm/h and an arsenic (As_2_) V/III ratio of ~15 and then to 525~540 °C to grow three layers of InAs QDs capped by a 4 nm InGaAs strain-reducing layer (SRL) and a 40 nm GaAs space, an uncapped QD layer for atomic force microscopy (AFM). The QD height was extracted from an AFM image for statistics of the size distribution [13]. The In deposition in each QD layer was divided into 4~8 circles, each with deposition step (2~4 s, rate: 0.05, 0.1 or 0.2 ML/s) and interrupt step (10 s) for sufficient migration, in an As_2_ pressure of 1.3 × 10^−6^ Torr, with the critical coverage θ_c_ for the island monitored by reflection high-energy electron diffraction (RHEED). The SRL in a proper In content forms *Dot-in-Well* to reduce In-Ga mixing and keep the QD height uniform and, also, to tune the QD wavelength. The in situ annealing was performed at the same As_2_ pressure by heating to >580 °C in ~6 min and then cooled back to the QD growth T (schematized in Figure 1a). The phase change is based on the fact that at a high T arsenic has a much higher vapor pressure than Ga [18] to reduce the residual surface arsenic, and the surface atom migration is fast for reconstruction. The annealing at different Ts form different amounts of β2 (as the nucleation site) among the β or α phases, offering a flexible phase preparation, as compared to the ‘stay-by’ to form the β/γ phase. Since the duration of the QD growth is <90 s, faster than the substrate T change (−10 °C/min) and the reconstruction phase change (tens of min), the annealing enables QD island at an optimal T and optimal reconstruction phase, β2 or β2 site-mixed α or β. The T mentioned here is the nominal T obtained in the electronic module. The PL performance was studied using a spectrograph equipped with a cooled InGaAs linear array detector: the room-T PL was measured by a multi-mode optical fiber beam splitter to introduce a laser (632.8 nm, 2 mW) and collect PL and a fiber probe on a sample micro-region of 62 × 62 μm^2^; the cryogenic-T PL was measured by confocal microscope (collection efficiency <5%) with a high-power 532 nm laser excitation (25 mW, focus on 4 μm^2^) and with the sample mounted on the cold finger (T = 4.5 K) in a closed-cycle helium cryostat. Their comparison reflects the carrier populations in QD discrete states. The QDs show two size-modes and the PL is mainly from large dots with the spectral FWHM related to its size distribution. The PL intensity at different sample Ts follows the Arrhenius equation I(T) = I_0_/[1 + Σ*A*_i_exp(−*E*_i_/*kT*)], where *E*_i_ is the QD thermal activation energy and *i* means different discrete levels. Figure 1 gives the phase diagram and structure models of the (2 × 4) reconstruction phases with descriptions of their influences on In adatom migration and nucleation.

## 3. Results and Discussion

**Figure 2:**
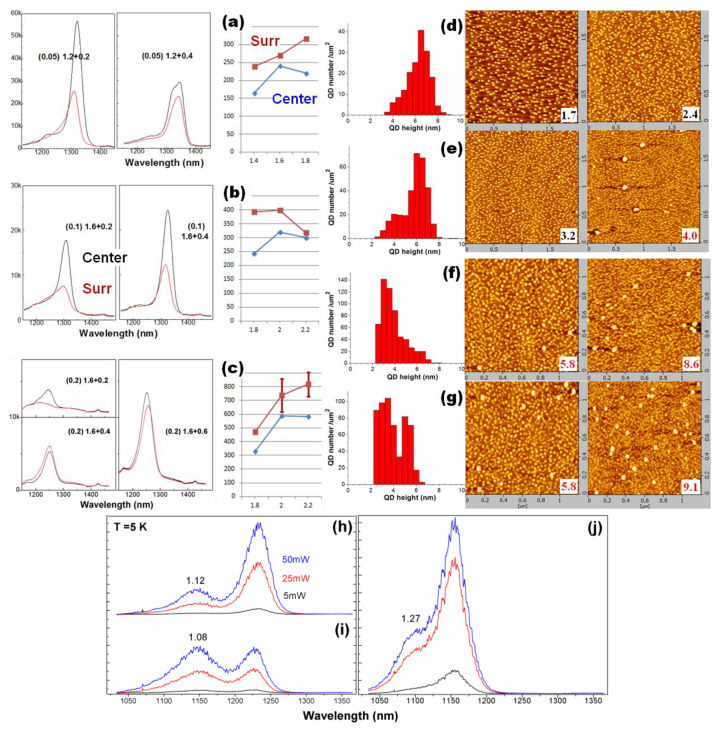
Room-T PL spectra and QD density (μm^−2^) as a function of In coverage, at rates of (**a**) 0.05, (**b**) 0.1 and (**c**) 0.2 ML/s. (**d**–**g**) AFM images (left: wafer center, right: Surr, density (×10^10^ cm^−2^) marked) and QD height statistics (wafer center) of the samples: (**d**) (0.05, 1.6), (**e**) (0.1, 2), (**f**) (0.2, 2), (**g**) (0.2 ML/s, 2.2 ML), size: 2 × 2 μm^2^ in (**d**,**e**), 1.2 × 1.2 μm^2^ in (**f**,**g**). For (**f**) and (**g**), at wafer Surr, the images with higher density were selected. **Bottom**: Excitation power-dependent T = 5 K PL spectra of QD samples (**h**) 2b2.0, (**i**) 2a1.4, and (**j**) 2c2.0. There are two spectral peaks. For the higher-energy peak, the slope (S) of excitation power-dependent intensity I = I_0_ × (P/P_0_)^S^ is marked. As reference, the slope for the large-dot ground-state (i.e., low-energy) peak is normalized to 1.

**Figure 3:**
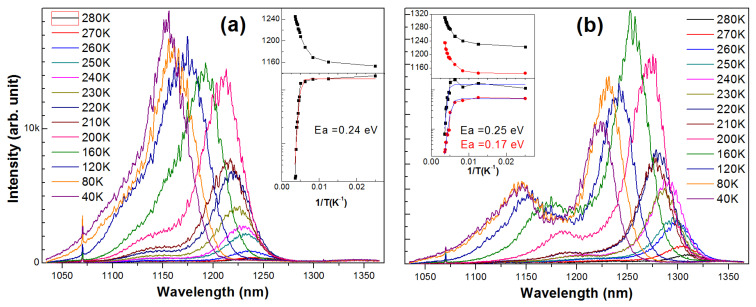
T-dependent PL spectra of QDs in sample 2c (**a**) and 4b (see Figure 4) (**b**), insets: PL intensity and peak energy (red: p-state, black: s-state) as a function of sample T in measurement, with Arrhenius fitting curve to extract Ea. Sharp line at 1064 nm is from 532 nm laser source.

Figure 2 presents three series of samples at rates of 0.05, 0.1 and 0.2 ML/s (QD densities of 1.7~2.4, 3.2~4.0 and 5.8~9.0 × 10^10^ cm^−2^, respectively). The sample 2a (Figure 2a) and 2b series (Figure 2b) were grown at 530 °C with ‘stay-by’; the sample 2c series (Figure 2c) was at 525 °C with annealing. As the In deposition amount raises, the QD density first increases and then decreases (with clusters). From the PL spectra it was found that for the rate of 0.05 ML/s the optimal amount is 1.4 ML (θ_c_: 1.2 ML) before forming bi-mode large QDs at 1.6 ML (larger spectral FWHM). For the rate of 0.1 (0.2) ML/s with slow migration, the optimal amount for a sufficient island is 2.0 ML (θ_c_: 1.6 ML). For the rate of 0.2 ML/s with limited migration (β2), as the In deposition raises from 2.0 to 2.2 ML, the QD density remains at 5.8 × 10^10^ cm^−2^ in the center while rising from (7.4 ± 1.2) × 10^10^ to (8.2 ± 0.9) × 10^10^ cm^−2^ at the Surr (the variation is related to the T distribution), with the highest one reaching 9.1 × 10^10^ cm^−2^ as presented. The dilute QD growth shows three critical coverages (θ_c_ −0.1, −0.07 and −0.01 ML) for large dot (height: 4~5, 7~11 and 12~18 nm) formation [13]; the QD ensemble here shows a continuous height distribution (2~9 nm) with more In coverage. The QDs showed two size modes and the room-T PL spectra are from large dots in the 2nd critical coverage. The spectral peak at 1.32 μm in samples 2a1.4 and 2b2.0 (shifting to 1.22~1.24 μm at T = 5 K, as Figure 2h,i shows) is from large dots at a height of 5~9 nm, >80% of all. The QD height decreases to ~7.4 nm after capping, as simulated in terms of their discrete levels [21]. For the high rate of 2.0 ML/s (i.e., the 2c series), the QDs show lower height and dense small dots (height: 2~4.5 nm). The room-T PL spectral peak at 1.25 μm (shifting to 1.16 μm at 5 K, Figure 2j) is from large dots at a height of 4.5~7.0 nm; the higher amount 2.2 ML greatly increases them (33% of all dots, 190 μm^−2^) for higher PL, with 7~8 nm-height QDs grouped into clusters. The coexisting clusters (more at the Surr) affect the PL performance of dense QDs little due to limited migration; while a fast migration at a low rate (sample 2a and 2b) shows QD PL seriously affected by cluster formation. In the 2c series, the PL spectra show wafer uniformity since the pre-grown annealing at 580 °C formed β2 sites on the whole wafer for preferential nucleation; the dense QDs show a PL spectral FWHM of 34 (41) meV at the wafer center (Surr), largely uniform (a lower or higher-T growth will increase the FWHM); the lower PL intensity than sample 2a is from random filling of photo-holes and electrons in dense QDs. At low T with complete filling in of large dots, there is a comparable maximal intensity (see T-dependent spectra, Figure 3). Compared to sample 2a and 4b with low-T PL emission at 1.22~1.24 μm from large dots at a height of 5~8 nm (Figure 2h,j), the dilute QDs show PL emissions at 1.22~1.24 μm (T = 77 K) correlated to QDs of AFM height 15~17 nm [13], i.e., there is more strain inside than in the dense QDs in 2a and 4b. In this learning, the shorter emission wavelength of dense QDs in the 2c series is mainly from their smaller height than in 2a and 2b (see QD height distribution), instead of strain accumulation. Large dots get the highest density (240 μm^−2^, 80% of all) in the rate of 0.1 ML/s (2b2.0). However, as the low-T PL spectra show (see Figure 2h–j), the dense QDs in the 2c series show more ground-state populations, i.e., more large dots formed, likely during the SRL capping. As Figure 3 shows, sample 4b (in the same low rate and low QD density as 2a) shows obvious p-state population and the s-state emission reduces at T < 160 K, related to the QD filling-induced ‘environment’ electric field or the population in small dots, which must be considered for devices. The excited state in the dense QDs (2c) is little filled as T varies or as excitation power raises, likely from their inter-dot coupling, desired for high injection and optimal emission in the ground state. For the growth rate of 0.1 ML/s, sample 2b2.0 shows similar PL spectra and wafer distribution as the rate of 0.05 ML/s (2a1.4); its spectral FWHM is 28.3 (34.1) meV at the wafer center (Surr), higher than 2a1.4 (24.6 meV, at the center); its greater number of large dots show more s-state transition in the low-T PL spectrum (Figure 2h). In Figure 3, the T-dependent PL spectra reflect comparable intensity for dense QDs in sample 2c and 4b at low T and QD thermal activation energy, ~0.24 eV for QD s-state and 0.17 eV for p-state, reflecting the QD electron level offset to the continuum conduction band [21] (meanwhile, the QD hole level offset to the continuum valence band is ~0.19 eV, promising for a high T_0_ in a laser diode). As the sample T reduces, the PL peak blue-shifts; with respect to the QD s-state, a larger shift as the T varies existed in p-state, reflecting more influence from the continuum band. For dense QDs (2c), there is a smaller energy shift in the QD p-state, which was related to its inter-dot coupling and mini-band formation possibly.

**Figure 4:**
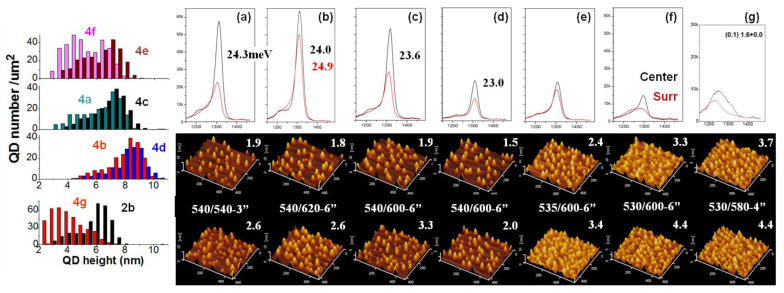
Annealing influence on QD growth at rates of 0.05 ML/s (**a**–**f**) and 0.1 ML/s (**g**), room-T PL spectra (black: center, red: Surr) and 0.45 × 0.42 μm^2^ AFM images (bird-view, upper: center, lower: Surr, density (×10^10^ cm^−2^) marked). Left: QD height statistics (at the center).

**Figure 5:**
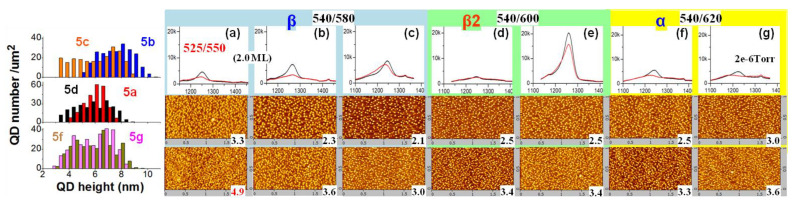
Annealing influence (β: 580 °C, β2: 600 °C, α: 620 °C) on QD growth at a rate of 0.2 ML/s. (**a**) (2.0, 525), (**b**) (2.0, 540), (**c**–**g**) (1.8 ML, 540 °C), (**e**) higher-In SRL, and (**g**) high arsenic during annealing. Room-T PL spectra (red: Surr, black: center) and AFM images (upper: center, lower: Surr, density (×10^10^ cm^−2^) marked). Left: QD height statistics.

Figure 4 explores the influence of different annealing Ts (T_an_) on the QD growth at a rate of 0.05 ML/s and at the optimal amount (1.4 ML) and growth T (T_gr_) of 540 °C. The PL peak is from large dots (height: 5~9 nm) in the spectral FWHM of ~24 meV. The smallest FWHM is 23.0 or 23.6 meV, obtained in 4d or 4c with β2 (T_an_ = 600 °C). Although the same nominal T was found for 4c and 4d, the real T was a little higher in 4d with more β2 for uniform island at both the center and Surr (see AFM image), forming QDs in lower density and smaller FWHM, with sharp tops and small bases, and with more strain accumulated for a low PL. In β2, as sample 4c–f (AFM images, PL spectra and QD height distribution) shows, the QD island is sensitive on growth T (T_gr_), i.e., a 5 °C increase will improve In migration greatly. The QD height distribution in the left reflects the QD growth: In sample 4f and 4e with lower T_gr_, the QD height is distributed at 2~9 nm equally with PL spectrum in a broad profile and peak at 1.3 μm from the large dots (height: 7~8 nm). In 4b and 4d with higher T_gr_, the QD height distribution changes and more large dots (height: 7~10 nm) form. In the β phase (4a,c and 2a), the QD growth shows similar height distribution as in 4b and 4d but at lower height of 3~9 nm. The 1-nm increase of the QD height in β2 is from preferential nucleation in the β2 nucleation sites (atomic stage). In α (T_an_ = 620 °C, 4b) or β (‘stay-by’, 4a) with improved migration for a sufficient island, it shows a high PL in a larger FWHM and the same density (1.9 (2.6) × 10^10^ cm^−2^ at center (Surr)) as sample 2a1.4. In the α phase (4b), the PL spectra are wafer-uniform: at the Surr, the high PL intensity, comparable to the center, is due to β2-mixed α phase to improve migration and nucleation for the uniform island of tall and large-base QDs (see AFM image) in FWHM of ~24.9 meV. Compared to this, the β phase shows diverse-size QDs at the Surr in lower T for migration. The T-related migration leads to the same thing in 4c and 4d. The wafer center showsa sufficient migration for island at T_gr_ ≥ 535 °C. Sample 4g (0.1 ML/s fast migration, T_an_ = 580 °C, 4 min, small-amount β2) shows diverse QDs (height 2~7 nm, see size distribution) in the center, of density 3.7 × 10^10^ cm^−2^ at a deposition amount of 1.6 ML (the same level is achieved at amount 2.0 ML in β phase, 2b) and spectral FWHM of 117 nm (two size modes, 2~5 nm and 5~7 nm in height, with spectra overlapped) (higher spectral FWHM and PL intensity with QD height covering 3~10 nm are expected when the In deposition amount increases to 2.0 ML); while it shows uniform QDs (FWHM ~68 meV) at the Surr in density of 4.4 × 10^10^ cm^−2^ and similar spectrum as 2c (i.e., lower T with slow migration for sufficient island on a small amount of β2 sites). Without the annealing or β2 site, it forms uniform QDs in the β phase at the same T_gr_ (2b in Figure 2, FWHM ~28.6 meV, large dots of height 5.5~8 nm, see height distribution). Sample 4f grown in 0.05 ML/s with T_an_ = 600 °C (more β2 sites) also shows diverse-size QDs at both the wafer center and Surr (see AFM images), uniform spectrum in the center (un-ripened), and a lower and broad spectrum in the Surr (more overlap of the QD base). The diverse-size QDs in sample 4g (center) shows little overlap of the QD base. In all, the growth of diverse-size QDs required β2 nucleation sites and a non-uniform In atom supply (i.e., more β2, lower rate, higher T_gr_), unlike the growth of uniform dense QDs (i.e., high rate, proper T_gr_ and proper β2 amount) with sufficient migration. For high rate, the high-T_gr_ growth often promoted migration to form QDs at a lower density than we expected, as the AFM images in Figure 5 show. In the β phase with a smaller amount of β2 sites (T_an_ = 550 °C), sample 5a also shows lower density QDs, of 3.3 (4.9) × 10^10^ cm^−2^ at the wafer center (Surr), and different PL intensities on wafer. In all, to form a QD density >5 × 10^10^ cm^−2^, it is better to use proper β2 nucleation sites at a proper T_gr_ to limit migration and increase the island. Unlike the precise calibration of T_gr_ each time by RHEED observation of the β-to-γ transition point, the pre-grown annealing (T_an_, duration) offers a universal modulation of the reconstruction phase on the whole wafer (β2-mixed β or α) with a large tolerance of wafer T variation.

Figure 5 explores the annealing effect on QD growth at a high rate. QDs were grown at 540 °C in sub-optimal amount and at a density of 2.5 (3.4) × 10^10^ cm^−2^ at the wafer center (Surr), as shown in Figure 5d–f. This were lower than that grown at 525 °C with the same amount (3.3 (4.7) × 10^10^ cm^−2^, see Figure 2c, the plot of QD density v.s. deposition amount). Different annealing Ts (±20 °C) define different reconstruction phases as indicated. In β2 (5d,e) or α (5f,g) that mixes with β2, it forms wafer-uniform PL-lacking QDs, with a broad size distribution (height 3~9 nm). In the β phase (5b,c) with T-sensitive migration on the wafer, the optimal deposition amount reduces at the high T_gr_: Sample 5c, at an amount of 1.8 ML, shows a sufficient island and PL-optimal QDs, wafer-uniform due to the mixed β2 (like the case in 2c, 5d,e), at a lower density of 2.1 (3.0) × 10^10^ cm^−2^ at the wafer center (Surr), and a broad height distribution, non-uniform island. Sample 5b at 2.0 ML shows a red-shift PL spectral peak in the center (QD height increased to 4.5~10 nm, see the distribution) and a degraded PL at the Surr (QD density increased to 3.6 × 10^10^ cm^−2^, with a high rate and high T for sufficient island). In β2 and α phases, the PL-optimal deposition amount is retained, independent of the higher T_gr_, as the PL spectra (similar to that grown at 525 °C with the same amount, 2c1.8) reflects. In the β2 phase (5d,e), with limited migration on the whole wafer, the PL is wafer uniform; the higher-In SRL in 5e greatly improved it since In-Ga mixing is reduced and the QD height is retained. In the α phase (5f,g), the increase of the arsenic pressure (2 × 10^−6^ Torr) during annealing (5g) shows a blue-shift PL spectral profile at the Surr with QD density increased, while a PL peak in the center with the QD density increased too, to 3.0 × 10^10^ cm^−2^, in the same height distribution as shown. The reason for the PL blue-shift at the Surr is the Ga-As bond formation and desorption in the form of Ga-As molecules in an arsenic atmosphere needed to break the Ga-Ga bond in the α phase and leave β2 with limited migration for island. In the wafer center at a higher T, the fast adatom migration forms a Ga-As-Ga bond (i.e., α transfer to β with migration unaffected), and there is always a PL peak from the QDs, independent of the arsenic pressure. In all, the high-T growth in β shows an island in advance (5c, 4g) from the mixed β2 sites; the high-T growth in α (5f, 4b) shows a transfer to β2 at the wafer Surr or β at the center, with an improved wafer-uniform QD island.

The PL performances of different density QDs is estimated in a formula considering the random filling of photo-electrons and holes in QDs: I ~ η[n + (n − 1)C(N − n,1) + (n − 2)C(N − n,2) + (n − 3)C(N − n,3)+…]/C(N,n) for N > n and ~ηN/n for N < n, where N denotes the QD number in a sub-μ-region 200 × 200 nm^2^, n the laser-excited photo-electron number, η the QD quantum yield, C(m,n) the combination of n electrons filling in m dots and I the PL intensity (i.e., spectral peak area). In this estimation, dense QDs in the 2c series maintains the highest η (PL performance). This is also reflected in the T = 5K PL spectra with the excited state filled (Figure 2h–j and Figure 3): sample 2c series maintains the highest PL (i.e., little-filled excited state); the samples grown at a rate of 0.05 ML/s with fewer large dots show an obvious population on the excited state (e.g., 2a shown in Figure 2i); the sample at 0.1 ML/s with more large dots shows less population on the excited state (2b). As the excitation power increased, the excited state is more populated, as Figure 2h–j shows.

## 4. Conclusions

In summary, we use pre-grown annealing to form less-arsenic reconstruction β2 and fabricate high-density uniform InAs quantum dots (QDs) on 3-inch GaAs substrates with wafer-uniform PL. The maximum density reaches 5.8 (8.2 ± 0.9) × 10^10^ cm^−2^ with a room-T PL spectral FWHM of 34 meV (41 meV) in wafer center (Surr), grown at a high rate and low T to limit In migration. Low rate growth at high T forms uniform QDs at a density of 1.9 × 10^10^ cm^−2^ and a spectral FWHM of ~23.6 meV. Both show wafer-uniform PL spectra, unlike the β phase (T-sensitive). Mediate rate at a low T forms uniform QDs in the β phase (3.2 × 10^10^ cm^−2^, FWHM ~28.6 meV) at the center and diverse QDs in β2 (3.7 × 10^10^ cm^−2^ and spectral FWHM ~117 nm). The pre-grown annealing provides a controllable modulation of the surface reconstruction and enables an optimal T to form dense wafer-uniform QDs. 

## Data Availability

The data that support the findings of this study are available from the corresponding author upon reasonable request.
